# Open questions on transition metals driving secondary thermal processes in atmospheric aerosols

**DOI:** 10.1038/s42004-021-00616-w

**Published:** 2021-12-14

**Authors:** Hind A. Al-Abadleh, Sergey A. Nizkorodov

**Affiliations:** 1 Department of Chemistry and Biochemistry, Wilfrid Laurier University, Waterloo, ON N2L 3C5 Canada; 2 Department of Chemistry, University of California Irvine, Irvine, CA 92697 USA

**Keywords:** Atmospheric chemistry, Spectrophotometry

## Abstract

Transition metals are increasingly recognized as key drivers in the formation and aging of light-absorbing organic aerosols, known as brown carbon, which impact the energy flux in the atmosphere. Here the authors discuss somewhat overlooked condensed phase chemical processes and identify research needs to improve our fundamental understanding of atmospheric aerosols and ultimately reduce modelling uncertainties of the direct and indirect effects of aerosol particles on the climate.

The physical and chemical properties of aerosol particles from primary and secondary sources^1^ influence radiative forcing (RF) directly through absorption and scattering of incoming radiation, and indirectly by acting as ice/cloud condensation nuclei and providing surfaces for chemical reactions that change the chemical balance of atmospheric gases^2^. The latest Intergovernmental Panel on Climate Change (IPCC) report^2^ shows that aerosol effective RF ranges from −0.4 to −2.1 Wm^−2^, while that of carbon dioxide (CO_2_) ranges from +1.8 to +2.2 Wm^−2^. These RF ranges reflect our current level of scientific understanding and clearly show that, while we are reasonably confident of the warming effect of CO_2_, there are still knowledge gaps in aerosol-cloud and aerosol-radiation interactions. A large fraction of this uncertainty comes from light-absorbing atmospheric particles that affect the radiative energy balance directly. While RF for non-absorbing sulfate aerosol ranges from −0.3 to −0.8 Wm^−2^, RF for black (i.e., soot) and organic aerosol is less certain, ranging from −0.4 to +0.4 Wm^−2^. The light-absorbing brown carbon aerosol is currently thought to include polycyclic aromatics, humic-like substances, biopolymers, etc.^3^, produced by a variety of primary and secondary processes. The chemical composition of aerosols evolves over atmospheric residence time because of physical and chemical aging processes^4^. Atmospheric and climate models are developed based on our current understanding of atmospheric composition and dynamics^5^. So, there is an urgent research need in *atmospheric aerosol chemistry* to address our knowledge gaps in two main areas: mechanisms of formation of light-absorbing compounds and pathways that lead to their aging.

## Aerosol chemical reactivity and physical properties

**Fig. 1:**
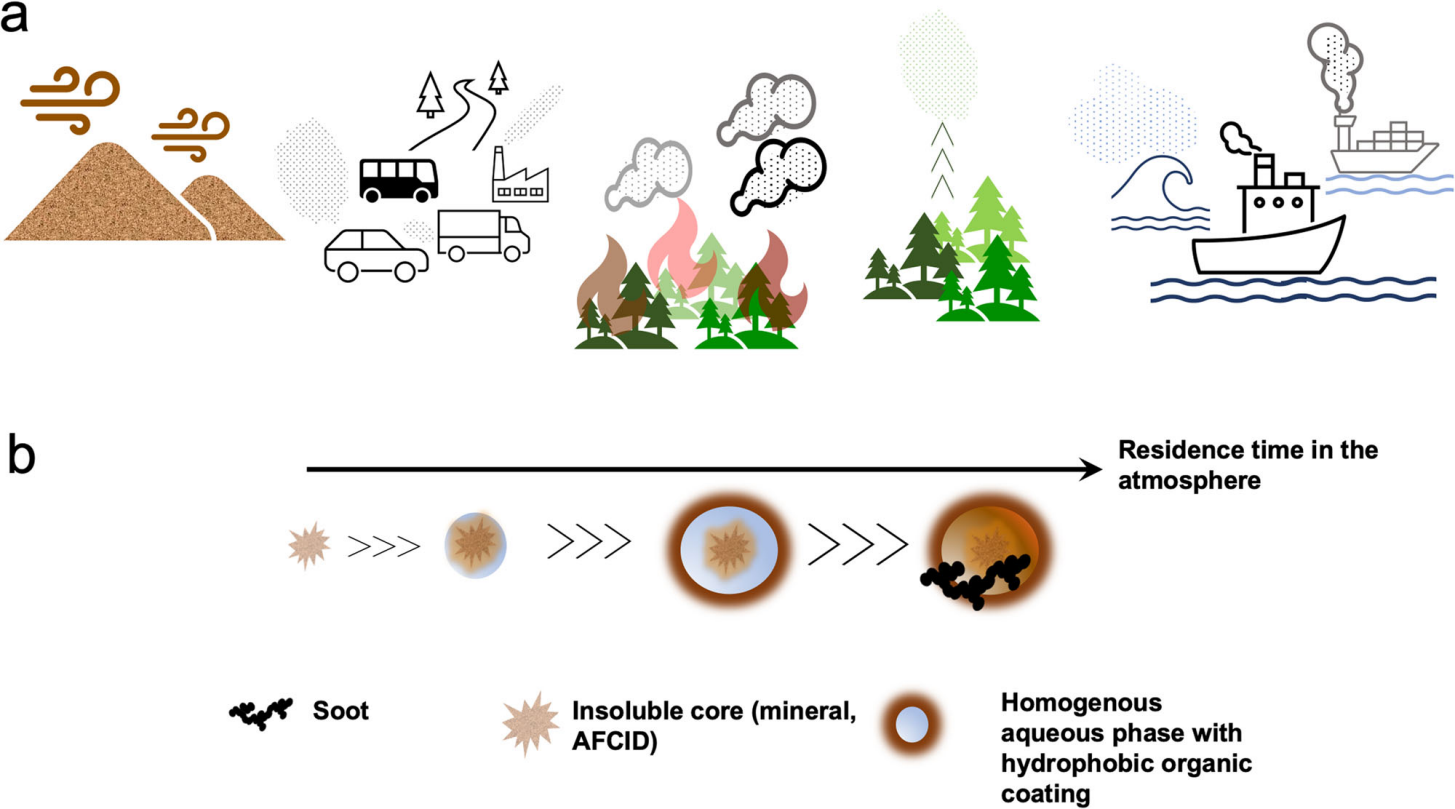
Schematic of the natural and anthropogenic sources of atmospheric aerosol and how long-range transport of mineral and anthropogenic fugitive, combustion, and industrial dust (AFCID) particles change their physicochemical properties. **a** Primary sources of atmospheric aerosol particles include mineral dust from natural dust storms, particulate matter from incomplete combustion processes from transportation and industrial activities, biomass burning organic aerosols (BBOA) from wild forest fires, secondary organic aerosol (SOA) particles that form in the atmosphere—represented by the vertical arrows—from the (photo)oxidation of biogenic volatile organic compounds (VOCs), sea spray from ocean/sea wave action, and particulate matter from ship emissions. **b** Schematic of the changes to mineral dust particle size, shape, morphology, hygroscopicity, and optical properties during their residence time in the atmosphere. These changes take place due to acid processing from heterogeneous reactions with acidic gases and VOCs, particle mixing with other aerosol particles such as soot, and multiphase reactions that include homogeneous reactions in the aqueous phase and liquid/solid interfacial reactions.

The term aerosol aging collectively refers to all the processes that change aerosol physicochemical properties during their atmospheric residence time, which can last up to two weeks (Fig. 1)^4^. These processes take place at the surface of the particles/droplets or within the aerosol condensed phase. Most studies of aerosol aging^6,7^ have focused on photochemical and free-radical-driven reactions, but not enough attention has been paid to redox processes that can readily occur in dark conditions, and do not require free radicals to start, but are instead initiated by transition metals. The extent and rates of these reactions are strongly influenced by aerosol water content which affects aerosol acidity and ionic strength^8,9^. In general, wet aerosol particles are highly acidic (pH < 2) and ionic strength was calculated to be in excess of 10 M, compared to droplets in rain, clouds, and fog (pH 2–7) with ionic strength between 6 × 10^−6^ and 6 × 10^−2^ M. In the presence of hygroscopic salts, variation in relative humidity (RH) affects the amount of aerosol liquid water (ALW) leading to phase transitions (deliquescence with increasing RH and efflorescence with decreasing RH). In multicomponent aerosol systems containing salts and organics, liquid–liquid phase separation (LLPS) takes place which changes aerosol mixing state and morphology^10^. Therefore, aging processes can happen over a range of conditions, from dilute solutions, to concentrated solutions, to surfaces, and solids.

## Metal-free formation of light-absorbing organic aerosol

Biomass burning is arguably the largest source of primary organic aerosol in the atmosphere, and some of the emitted components (such as nitrophenols) are light-absorbing^11^. Secondary organic aerosol (SOA) produced by gaseous phase, aqueous phase, and multiphase oxidation of VOCs contribute even more particulate organics^12^. Several groups of VOCs are known to produce light-absorbing oxidation products, and this includes oxidation of aromatic hydrocarbons in presence of nitrogen oxides, as well as oxidation of heterocyclic (e.g., furans^13^) and substituted (e.g., phenols^14^) aromatics. In addition, thermal reactions of reduced nitrogen compounds, such as ammonia and amines, can produce secondary light-absorbing products in reactions with carbonyls^3^. These ammonia/amine + carbonyl reactions tend to be relatively slow, and the absorption coefficients, as well as photochemical stability of the reaction products, are generally too low to have an effect on climate^15^. Relevant to this discussion, none of the processes discussed in this paragraph require transition metals.

## Sources, concentrations, and chemical reactivity of transition metals in aerosols

During natural storms^16,17^, mineral dust particles top the list of atmospheric particulate mass loading followed by sea salt, sulfate, black and organic carbon. Human activities due to rapid urbanization and industrialization are also responsible for the emissions of an emerging class of unconventional mineral dust referred to as AFCID^18^, which largely contributes to the harmful fine and ultrafine particulate matter^19–21^. These dust particles contain transition metals such as iron (Fe) and copper (Cu)^18^. Iron is the most ubiquitous transition metal in dust particles because hematite (α-Fe_2_O_3_) is the third most abundant mineral by mass in the Earth’s crust, following silica (SiO_2_) and alumina (Al_2_O_3_). Long-range transport and atmospheric processing provide ample and frequent opportunities for dust particles to mix with organic and inorganic components of biomass burning smoke^22,23^. Heterogeneous chemistry in these plumes increases the solubility of iron-containing minerals depending on their composition, morphology, and amount of ALW^16,24–26^. Dissolved iron concentrations in rain, fog, snow, and cloud waters were reported to be location-dependent and varying by over four orders of magnitude, from 0.1 to 10^3^ μM^27^, with a typical concentration of dissolved Fe(III) of 1 μM in 20 μm cloud droplets. Using the typical lower limit of iron solubility of 5%, with a typical ALW content of 6 × 10^−8^ L m^−3^ of air and iron concentrations in fine particles of 331–1640 ng m^−3^ from across China^28^, dissolved iron in ALW would range from 5–24 mM. Therefore, the reactivity of dust particles is controlled by soluble and insoluble iron species, which under acidic conditions existing in atmospheric aerosol particles, can catalyze chemical processes leading to secondary organic and inorganic products^27,29–31^.

In our laboratories, we conducted experiments to address research questions about the role of iron in the aging of multicomponent aerosol systems using bulk and surface sensitive analytical and materials characterization tools. We used hematite and Arizona test dust (AZTD) as sources of insoluble Fe(III) to mimic chemistry in freshly-emitted dust, and iron chloride as a source of soluble Fe(III) cations to highly aged dust. We also used aromatic and surface-active phenolic compounds identified in biomass burning organic aerosols (BBOA) extracts, as well as aliphatic dicarboxylic acids commonly formed in VOCs oxidation as organic reactants. A comprehensive review of our results was recently published^4^, which highlighted the efficient formation of soluble and insoluble light-absorbing organic products, catalyzed by iron chemistry in the dark with phenolic compounds and dicarboxylic acids. What is remarkable about the reaction products is that they are highly conjugated and have mass-normalized absorption coefficients rivaling those of soot, the strongest carbonaceous light absorber in the atmosphere^4,32^. The abiotic oxidative polymerization of phenols and metal-catalyzed polymerization of dicarboxylic acids are efficient pathways taking place under a wide range of reaction conditions in real aerosol systems. These conditions include variable atmospheric aerosol mixing states (i.e., homogeneous solutions versus slurries of iron-containing oxides and dust) and chemical composition that affect the organic to inorganic mass ratio, pH, and ionic strength. We found that secondary particle formation in solution from these iron-driven reactions is not completely suppressed by the presence of competing iron ligands such as sulfate, nitrate, or dicarboxylic acids with two to five carbon atoms (C2-C5), or under UV-irradiated conditions. Dissolved oxygen is a key ingredient in the abiotic oxidative polymerization reaction mechanism because it leads to the formation of reactive oxygen species (ROS) in situ that drives the cycling between Fe(II) and Fe(III). Functionalized phenolic compounds that easily donate electrons to this redox chemistry and aliphatic diacids that strongly complex Fe(III) are the ones that showed the fastest reaction rates. Both types of organic compounds are common in the atmospheric environment.

Moreover, mineral dust particles are more efficient ice nuclei than organic aerosol particles and hence play a major role in cloud formation and lifetime^16^. Chemical aging due to heterogeneous reactions with reactive gas-phase molecules affects the hygroscopicity and the ice nucleation activity of mineral dust due to changes in aerosol acidity and salt content^16^. Despite progress in this area, the effect of organic coatings on the ice nucleation ability of mineral dust is far from complete, especially at a molecular level. We showed from ice nucleation studies using AZTD that black polymeric products, formed over a wide pH range, did not significantly impact ice nucleation or block ice nucleation sites, whereas, increasing pH decreased the ice nucleation ability of AZTD. These results clearly show that iron-catalyzed chemistry can be occurring on relevant time scales and at relevant concentrations of iron and organic reactants, leading to products with impressively strong absorption cross sections and minimum impact on ice nucleation activity of iron-containing aged dust.

## Outlook

Reducing the uncertainty associated with aerosol–radiation and aerosol–cloud interactions in atmospheric and climate models necessitates the incorporation of chemical processes driven by metals, such as iron, in multicomponent aerosol systems, particularly under aerosol-relevant conditions characterized by high acidity, ionic strength, and solute:solvent ratio. The open research questions centre around the effect of fuel type and burning conditions on BBOA chemical composition and the reactivity of their aqueous extracts with iron, the kinetics of redox cycling in metal-containing aerosol systems and how they are affected by ROS species, the kinetics of metal-catalyzed reactions that produce brown carbon in suspended aqueous microdroplets, and the influence of soluble and insoluble secondary organic products on the aerosol multiphase chemistry. In addition, future research should investigate the extent of iron solubilization in mineral dust/BBOA mixtures under the aforementioned aerosol-relevant conditions including salting-out effects of organics due to increased ionic strength. We believe that progress in this area will require a comprehensive understanding of the effect of iron and other transition metals on the physical properties of the organic components of aerosol, such as phase, mixing state, viscosity, absorption coefficient, hygroscopicity, and ice nucleation. We hope that this brief article will inspire additional experiments and modeling studies on this important topic. A possible laboratory experiment to probe some of these properties would be to use mineral dust particles as seeds for growing SOA from BBOA emissions, collecting the mixed particles and tracking their properties over long aging times under different environmental conditions. A possible field experiment would be to contrast properties of BBOA smoke produced during dusty conditions and dust-free conditions. Finally, modeling studies should include multiphase reactions for iron that lead to increased solubilization and the formation of light-absorbing products in the dark.
